# A New Module in Neural Differentiation Control: Two MicroRNAs Upregulated by Retinoic Acid, miR-9 and -103, Target the Differentiation Inhibitor ID2

**DOI:** 10.1371/journal.pone.0040269

**Published:** 2012-07-25

**Authors:** Daniela Annibali, Ubaldo Gioia, Mauro Savino, Pietro Laneve, Elisa Caffarelli, Sergio Nasi

**Affiliations:** 1 Consiglio Nazionale delle Ricerche - Istituto di Biologia e Patologia Molecolari (CNR – IBPM), Dipartimento di Biologia e Biotecnologie, Università Sapienza, Roma, Italia; 2 Dipartimento di Biologia e Biotecnologie, Università Sapienza, Roma, Italia; 3 Center for Life Nano Science @Sapienza, Istituto Italiano di Tecnologia, Università Sapienza, Roma, Italia; Instituto de Medicina Molecular, Portugal

## Abstract

The transcription factor ID2 is an important repressor of neural differentiation strongly implicated in nervous system cancers. MicroRNAs (miRNAs) are increasingly involved in differentiation control and cancer development. Here we show that two miRNAs upregulated on differentiation of neuroblastoma cells – miR-9 and miR-103 – restrain ID2 expression by directly targeting the coding sequence and 3′ untranslated region of the ID2 encoding messenger RNA, respectively. Notably, the two miRNAs show an inverse correlation with ID2 during neuroblastoma cell differentiation induced by retinoic acid. Overexpression of miR-9 and miR-103 in neuroblastoma cells reduces proliferation and promotes differentiation, as it was shown to occur upon ID2 inhibition. Conversely, an ID2 mutant that cannot be targeted by either miRNA prevents retinoic acid-induced differentiation more efficient than wild-type ID2. These findings reveal a new regulatory module involving two microRNAs upregulated during neural differentiation that directly target expression of the key differentiation inhibitor ID2, suggesting that its alteration may be involved in neural cancer development.

## Introduction

The Helix-Loop-Helix (HLH) transcription factor ID2 (Inhibitor of DNA binding-2) belongs to a small family of proteins (ID1-4) with key roles in developmental processes [1]. They usually promote proliferation and prevent differentiation. IDs associate to ubiquitous bHLHs and negatively regulate formation of homo- or heterodimeric DNA binding bHLH complexes [1]. IDs can also bind to PAX and ETS factors [2], [3], [4], and ID2, in particular, was shown to interact with the retinoblastoma protein RB and with HES1 [5], [6], [7]. ID protein expression is usually turned off upon differentiation and is very limited in normal adult tissues. IDs are aberrantly produced by tumour cells and tumour endothelium [8], and are considered targets for cancer therapy [9], [10], [11].

In nervous system development, ID2 is expressed in neural precursor cells and helps maintaining the neural stem cell pool by inhibiting precocious neurogenesis [10], [12], [13], [14]. Decreased ID2 expression and cytoplasmic sequestration promote neural differentiation [10]. ID2 is widely expressed as well in adult neural progenitor cells and represses their differentiation, but it is also present in a subset of post-mitotic neurons of the adult nervous system and it is required for differentiation of some neuronal subtypes [15].

An increased ID2 expression accompanies tumorigenesis in the nervous system [6], [8], [9], [10]. Specifically, ID2 plays a key role in proliferation of glioma stem-like cells [16], it supports tumour cell migration [17], and it is frequently upregulated in neuroblastoma, a childhood tumour arising from aberrant development of neural crest cells [7], [9], [18]. ID2 protein expression, intracellular localisation and stability are downregulated when neuroblastoma cell lines are induced to differentiate [19], [20], [21]. Therefore, ID2 may be implicated in maintaining the tumorigenic properties, as was indicated by previous work on an ID dominant interfering HLH domain named 13I [22]. Ectopic expression of the 13I protein in neuroblastoma cells triggered growth arrest, promoted differentiation, and enhanced the action of retinoids – such as all-trans retinoic acid (RA) –, agents that induce neural differentiation and are used in NB therapy [10], [20], [23].

In light of all this, it is important to elucidate the mechanisms controlling ID2 expression. ID2 production is known to be controlled by extrinsic signals such as Bmp and Wnt that act trough transcription factors like p53, which represses ID2 transcription, and N-Myc, which upregulates it [6], [13], [16], [24], [25]. Aside from transcription factors, critical regulators of differentiation and tumorigenesis include microRNAs (miRNAs) – small RNAs that suppress gene expression at the post-transcriptional level upon interaction with target mRNAs [26], [27]. Neural differentiation is accompanied by the induction of several miRNAs that are thought to have a modulatory role, by targeting mRNAs of important regulators of differentiation [28], [29], [30].

We asked whether microRNAs directly targeted the *ID2* mRNA, and we especially focused on those known to be upregulated by retinoic acid. We have identified two microRNAs – miR-9 and miR-103 – that are upregulated by RA in neuroblastoma cells, directly inhibit ID2 expression, impair proliferation and trigger differentiation. We propose that the ID2, miR-9 and miR-103 module is a component of neural differentiation control that might be targeted for promoting differentiation of neural cancer cells.

## Results

### miR-9 and miR-103 target *ID2* mRNA

**Figure 1:**
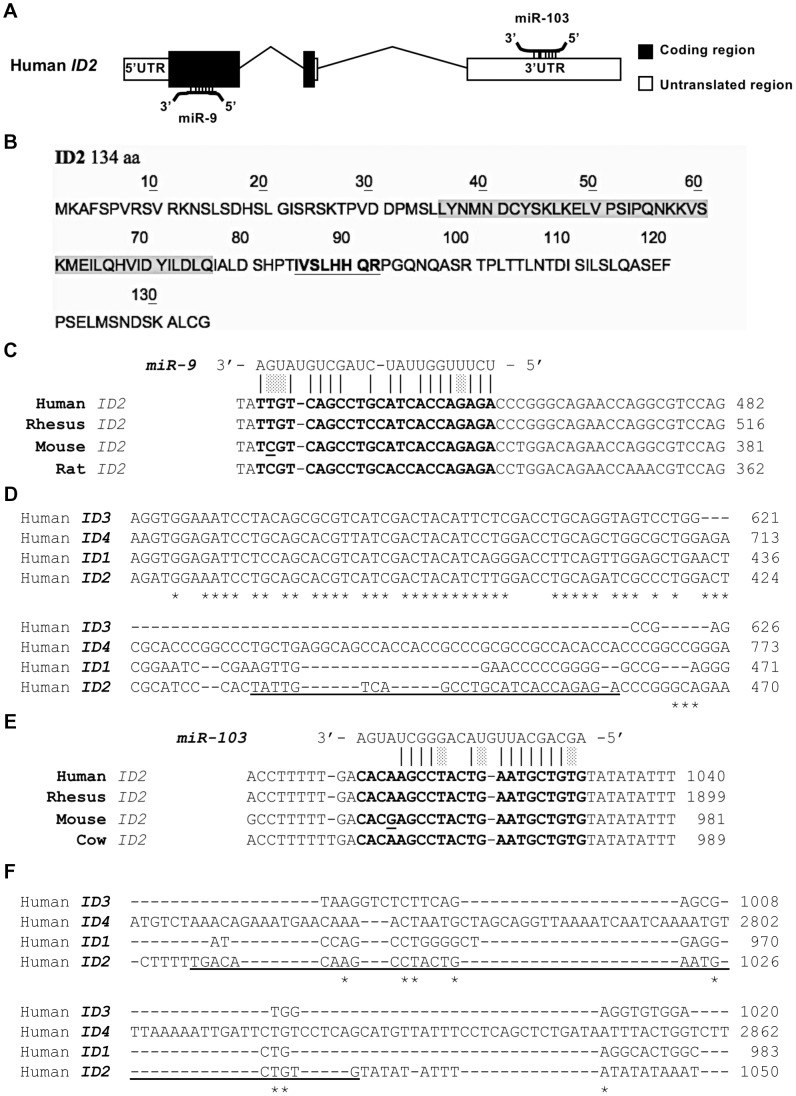
*In silico* analysis suggests that *ID2* mRNA may be recognized by miR-9 and miR-103. (**A**) Outline of the *ID2* gene and putative miR-9 and miR-103 binding sites in the first coding exon and 3′UTR region, respectively. (**B**) ID2 amino acid sequence showing the region corresponding to the putative miR-9 target sequence in *ID2* mRNA (bold, underlined) and the HLH domain (amino acids 36–76, highlighted). (**C**) Predicted duplex formation between human *ID2* mRNA and miR-9, and sequences of the putative miR-9 binding site and surrounding regions within the *ID2* coding regions of human, Rhesus monkey, mouse and rat. Nucleotide changes are underlined. (**D**) DNA sequence alignment of *ID1-4* coding regions indicates that the putative miR-9 target site (underlined) is exclusively present within *ID2*. (**E**) Predicted duplex formation between *ID2* 3′UTR and miR-103, and sequence of the putative binding site within the 3′UTRs of human, Rhesus monkey, mouse and cow. Nucleotide changes are underlined. (**F**) Alignment of 3′UTR sequences of the four *ID* genes (*ID1-4*) shows that the putative miR-103 recognition site (underlined) is present exclusively in *ID2*. Shades in the alignments of panels C and E represent wobble base pairs. Asterisks in panels D and F mark conserved nucleotides.

We performed a bioinformatic analysis to identify microRNAs recognizing *ID2* mRNA (accession number NM_002166.4), focusing on the set upregulated upon retinoic acid treatment of the neuroblastoma cell line SK-N-BE [29]. We first searched through miRNA databases by prediction methods – miRanda, PicTar, TargetScan and miRNAmap – designed to detect sites in mRNA 3′UTRs, the classical targets of miRNA action [26], [27]. By miRNAmap, which combines predictions from different algorithms, we identified a putative binding site for miR-103a – from now on called simply miR-103 – in the 3′ untranslated region of *ID2* mRNA (Fig. 1A). However, several studies have demonstrated that miRNAs bind extensively to coding sequences as well [31]. To take this possibility into account, we employed rna22, a pattern-based methodology that effectively detects binding sites in any mRNA location [32]. By rna22 – which requires that the RNA sequences to analyse be loaded by the user – we investigated the presence in *ID2* mRNA of putative binding sites for each single miRNA regulated by retinoic acid in SK-N-BE cells. We identified a putative miR-9 binding site in the *ID2* mRNA coding sequence (Fig. 1A) and confirmed the putative binding of miR-103 to the 3′UTR. The putative miR-9 target shows a perfect match with the miR-9 seed region, an overall match of 19 out of 23 nucleotides (including wobble G-U base pairs), and a folding energy of −27.7 Kcal/mol. It is remarkably well conserved in mammals (Fig. 1C). The miR-9 site is located outside the *HLH* domain – which is highly homologous among the four ID proteins – and is missing in *ID1*, *ID3*, and *ID4* mRNAs (Fig. 1B, 1D). The putative miR-103 binding site in the 3′UTR of *ID2* mRNA shows a perfect match with the miR-103 seed region, an overall match of 15 out of 23 nucleotides and a folding energy of −26.1 Kcal/mol. It is also well conserved in mammals and *ID2* specific (Fig. 1E, 1F).

Both miR-9 and miR-103 are induced by retinoic acid in neuroblastoma cells [29]. miR-9 is highly neuro-specific and regulates development of neural tissues [33]. It is induced during differentiation of progenitor cells to neurons and astrocytes, mediating neurogenesis [28]. miR-9 is encoded by three loci – *miR-9-1*, *-2*, and *-3*. Transcription of the first two in neuroblastoma cells is turned on by RA and inhibited by REST – a repressor of neural differentiation genes [34], [35]. miR-9-3, instead, does not respond to RA [35] but can be activated by N-Myc [36]. miR-103 is more widely expressed. It is modulated during neurogenesis [37] and has a role in regulating neuropathic pain by controlling neuronal calcium channel expression [38]. This miRNA is also key regulator of metabolism, with a role in controlling insulin and glucose homeostasis [39].

**Figure 2:**
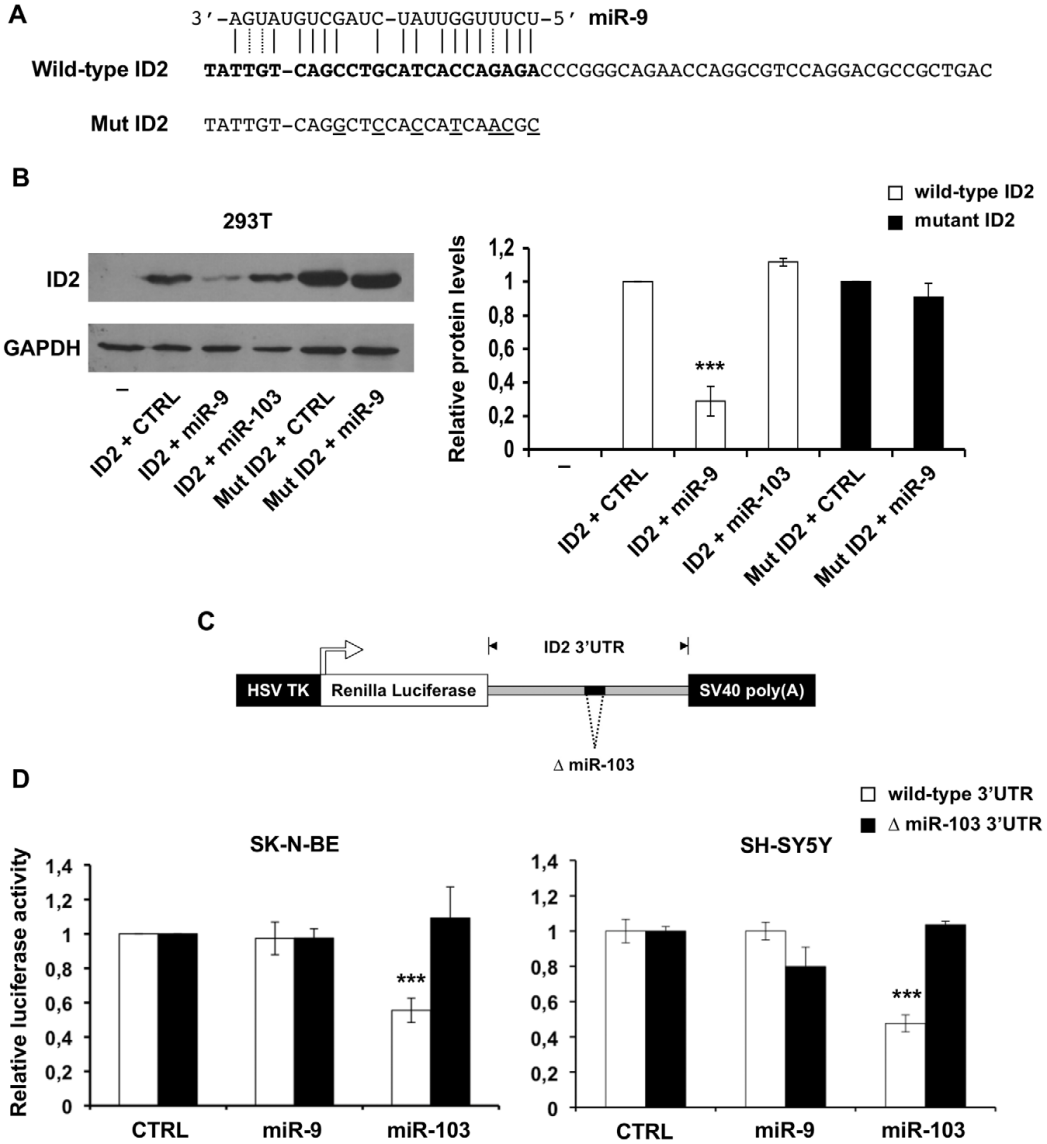
*ID2* mRNA is recognized by miR-9 and miR-103. (**A**) Duplex formation between miR-9 and the *ID2* coding region and sequence of mutated miR-9 recognition site present in Mut ID2. (**B**) Representative immunoblotting of ID2 in 293T cells transfected with vectors expressing the ID2 coding sequence - but not the 3′UTR - wild type or mutated in miR-9 recognition site (Mut ID2), together with vectors expressing miR-9, miR-103 or control vector (CTRL). Lane – represents untransfected cells. GAPDH was used as loading control. The histogram shows the relative quantities of ID2 and Mut ID2, as compared to cells transfected with control plasmids. (**C**) Luciferase reporter constructs harbouring the *ID2* 3′UTR or a mutant version carrying a deletion in the putative miR-103 target site (ΔmiR-103). (**D**) Luciferase activity (Firefly/Renilla ratio) of wild-type (white bars) and mutant (ΔmiR-103, black bars) *ID2* 3′UTR reporter gene in SK-N-BE (left) and SH-SY5Y (right) cells transfected with the miR-9 expressing vector, the miR-103 expressing vector or control (CTRL). Data are presented as mean values ± SD from at least three different experiments. ***: p-value<0.001.

We made use of ectopic miR-9 and miR-103 expression to validate the bioinformatic predictions. To determine whether miR-9 targeted the *ID2* coding region directly, we transfected the miR-9 vector together with expression plasmids containing wild type or mutated *ID2* coding region, but not 3′UTR, into 293T cells. We found that miR-9 decreased by about 70% ID2 expression driven by the wild-type coding region but not by a mutant one carrying altered nucleotides in the miR-9 site (Fig. 2A, 2B). ID2 expression driven by the coding region was unaffected by miR-103, as expected (Fig. 2B). To determine whether the 3′UTR was targeted by miR-103, we performed reporter assays upon co-transfection of miR-103 and luciferase reporters fused to wild type and mutant *ID2 3′UTR* (Fig. 2C, 2D). miR-103 ectopic expression decreased by 50% the activity of the luciferase reporter fused to wild-type *ID2* 3′UTR but not to a mutant one carrying a deletion of the miR-103 recognition site (Figure 2C, 2D). Activity of the 3′UTR reporter was unaffected by the miR-9 expressing plasmid.

Hence, the observed down-regulation of ID2 by miR-9 depends directly on a cognate recognition site in the coding region of *ID2* mRNA and does not involve the 3′UTR. Conversely, the down-regulation by miR-103 depends directly on a recognition site in the 3′UTR and does not involve the coding region.

### miR-9 and miR-103 downregulate ID2 in neuroblastoma cells

**Figure 3:**
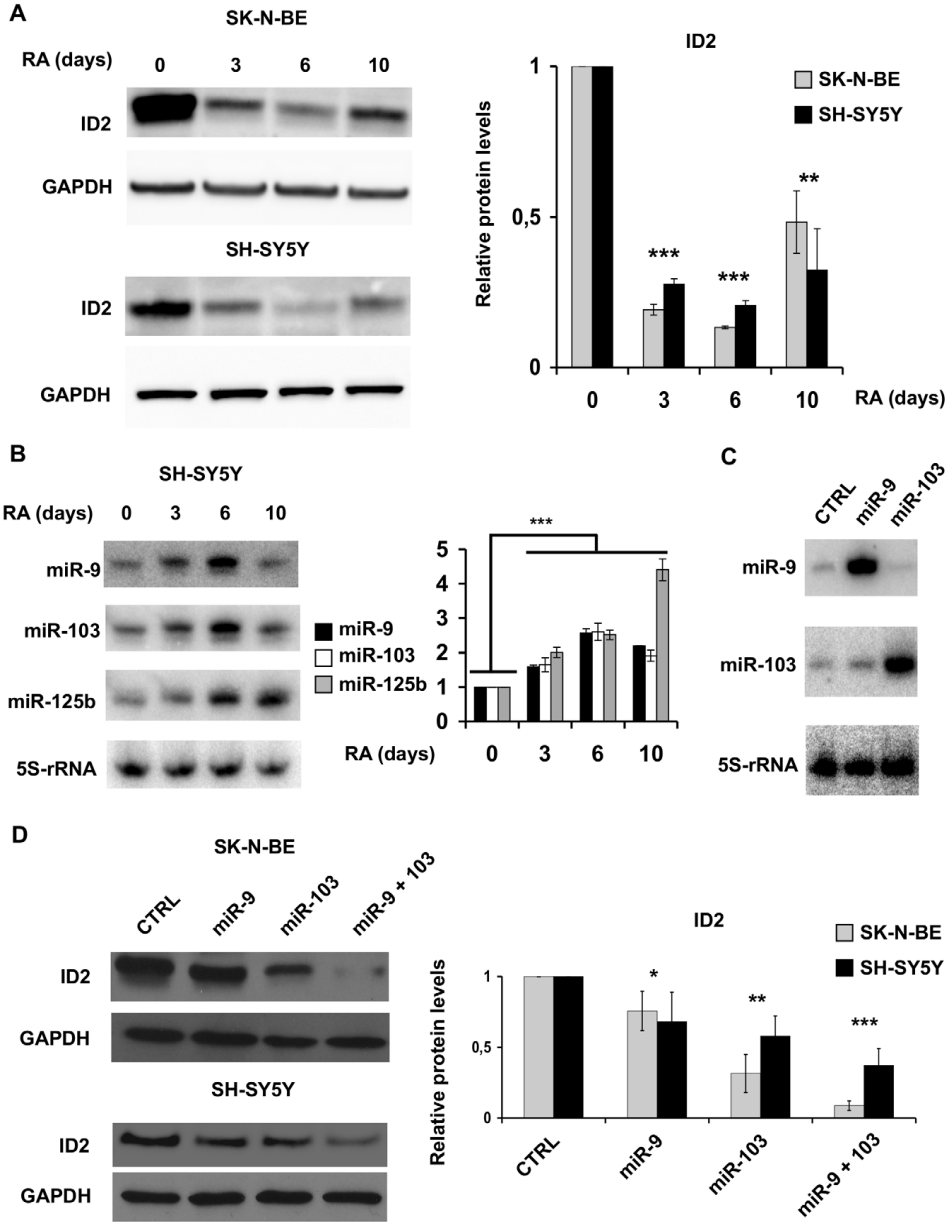
ID2 expression is inversely correlated to miR-9 and miR-103. Ectopic expression of miR-9 and miR-103 decreases ID2. (**A**) Immunoblotting of ID2 in SK-N-BE (upper panel) and SH-SY5Y (lower panel) cells treated with RA for 3, 6 and 10 days. The densitometric analysis on the right shows the relative amounts of ID2 versus untreated cells (0 time point), set to a level of 1. GAPDH was used as loading control. (**B**) Northern blotting of miR-9, miR-103 and miR-125b in SH-SY5Y cells treated with RA for the indicated times. The histogram shows the relative quantities of microRNAs versus the 0 time points set to a value of 1. 5S-rRNA was used as loading control. (**C**) Northern blotting of miR-9 and miR-103 in SK-N-BE ectopically expressing the single miRNAs. Cells transfected with an unrelated 21 nucleotide long RNA (CTRL) were used as control. (**D**) Representative immunoblotting of ID2 in SK-N-BE (top) and SH-SY5Y (bottom) cells ectopically expressing miR-9, miR-103, the two together, or the control vector (CTRL). GAPDH was used as loading control. Data in the histogram show the relative quantities of ID2 versus control cells. Data are presented as mean values ± SD from at least three different experiments. *: p-value<0.05; **: p-value<0.01; ***: p-value<0.001).

miR-9 and miR-103 are part of a small set of miRNAs upregulated upon retinoic acid treatment of the neuroblastoma cell line SK-N-BE, concomitantly with cell differentiation [29]. On the contrary, ID2 is down regulated by retinoic acid in neuroblastoma cell lines such as SH-SY5Y and others [19], [20]. This suggested that miR-9 and miR-103 up-regulation in differentiating neuroblastoma cells might be implicated in the ID2 decrease. To investigate this point, we compared the expression of ID2 with that of the two miRNAs in SK-N-BE and SH-SY5Y neuroblastoma cell lines [40], representing different cell types derived from such tumour [41], [42]. SH-SY5Y cells have wild type P53 and a single *N-MYC* copy, whereas SK-N-BE cells harbour mutant P53 and overexpress N-MYC due to gene amplification. ID2 was present in substantial amounts in both cell lines – at higher level in SK-N-BE – and its level decreased upon RA treatment (Fig. 3A). miR-9 and miR-103 expression, instead, similarly increased upon RA treatment in both cell lines ([29] and Figure 3B). The increased expression of miR-9 and miR-103 at 3 and 6 days of RA treatment was matched by a strong ID2 decrease, and the partial recovery of ID2 expression at 10 days was concomitant with miR-9 and -103 decrease (Figure 3B). The expression time course of the two microRNAs is shown for SH-SY5Y cells only (Fig. 3B) as it was previously reported for SK-N-BE cells [29]. miR-125b – another microRNA upregulated by RA [29] – showed a different behaviour (Figure 3B). This suggested that ID2 synthesis might be regulated by miR-9 and miR-103 and respond to altered miR-9 and -103 levels. To test this hypothesis, we ectopically expressed the two microRNAs in SK-N-BE and SH-SY5Y cells and measured endogenous ID2 expression by immunoblotting (Fig. 3C, 3D). Both miR-9 and miR-103 caused a significant decrease of the ID2 signal (Fig. 3D). Their combined expression caused a larger ID2 decrease in SK-N-BE versus SH-SY5Y cells, probably due to the different expression levels of ID2 in the two cell lines. miRNAs can mediate translational repression or mRNA degradation [43]. We found that miR-9 and miR-103 overexpression did not alter *ID2* mRNA levels (Figure S1), indicating that they act by repressing translation rather than affecting *ID2* mRNA stability.

Our findings show that ID2 is controlled at the post-transcriptional level by two miRNAs that are induced by RA and target *ID2* mRNA.

### miR-9 and miR-103 inhibit proliferation and promote neuroblastoma cell differentiation

ID protein inhibition by the dominant interfering 13I protein in two neuroblastoma cell lines reduced their proliferation and promoted differentiation [22]. This was mostly due to the inhibition of ID2, the most abundant ID protein in those cells [22]. We have shown that miR-9 and miR-103 repress ID2 production. Consequently, if ID2 inhibition is significant for their function, overexpression of the two microRNAs should mimic, at least partly, the effects observed upon 13I expression in neuroblastoma cells. To clarify this aspect, we asked whether ectopic expression of miR-9 and miR-103 affected neuroblastoma cell proliferation, differentiation marker expression, and neurite formation as it was shown to occur on ID2 inhibition by the 13I protein [22].

**Figure 4:**
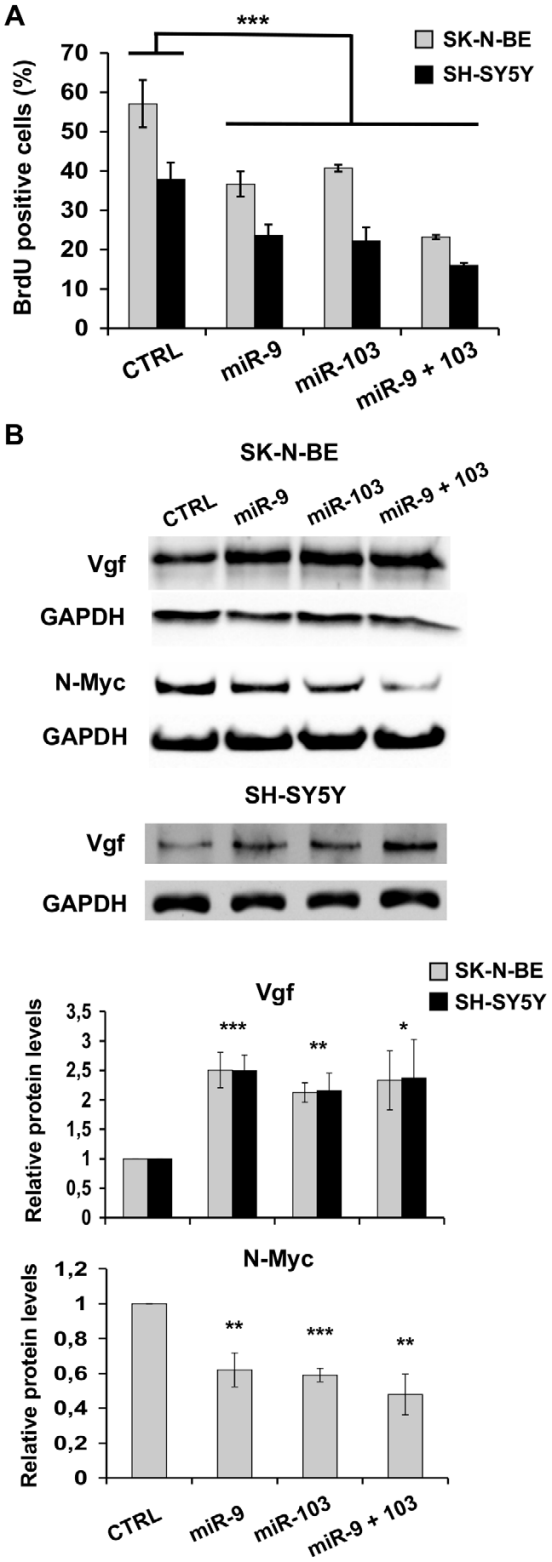
miR-9 and miR-103 restrain proliferation and affect differentiation marker expression. (**A**) BrdU incorporation assay in SK-N-BE (grey bars) and SH-SY5Y (black bars) cells transfected with plasmids expressing miR-9, miR-103, a combination of the two, or control (CTRL). Values represent means ± SD of three independent transfections. (**B**) Immunoblotting of Vgf and N-Myc in SK-N-BE (upper panel) and SH-SY5Y (lower panel) cells transfected with vectors expressing miR-9, miR-103, the two together or control (CTRL). GAPDH was used as loading control. The histograms display the relative quantities (means ± SD) of Vgf and N-Myc versus control cells, from at least three different experiments. *: p-value<0.05; **: p-value<0.01, ***: p-value<0.001.

Concordantly with the observations regarding 13I protein expression [22], we found that BrdU incorporation dropped by 40% following miR-9 and miR-103 ectopic expression in SK-N-BE and SH-SY5Y cells (Fig. 4A). ID2 downregulation by the two microRNAs might mediate, at least partly, this reduction, for instance by restoring RB control on proliferation. We then analysed the expression of two proteins modulated during NB cell differentiation: N-Myc, which is downregulated [44], and Vgf – a neuropeptide precursor induced by neurotrophins [45] –, which is upregulated by RA in SK-N-BE cells [46], [47]. Induction of *vgf* expression involves binding of a bHLH protein complex to the promoter [45]. VGF was increased 2–2.5 fold upon miR-9 and miR-103 ectopic expression in SK-N-BE and SH-SY5Y cells (Fig. 4B). Conversely, ID2 overexpression hampered the VGF increase observed after RA treatment (Figure S2), indicating that the effect of RA on expression of VGF involves the inhibition of ID2. N-Myc expression was examined in SK-N-BE cells, which display a high expression level due to gene amplification; its level was decreased by 40–50% upon miR-9 and miR-103 ectopic expression (Fig. 4B). Therefore, miR-9 and miR-103 hinder proliferation and promote differentiation marker expression, mimicking the effects of ID2 inhibition.

**Figure 5:**
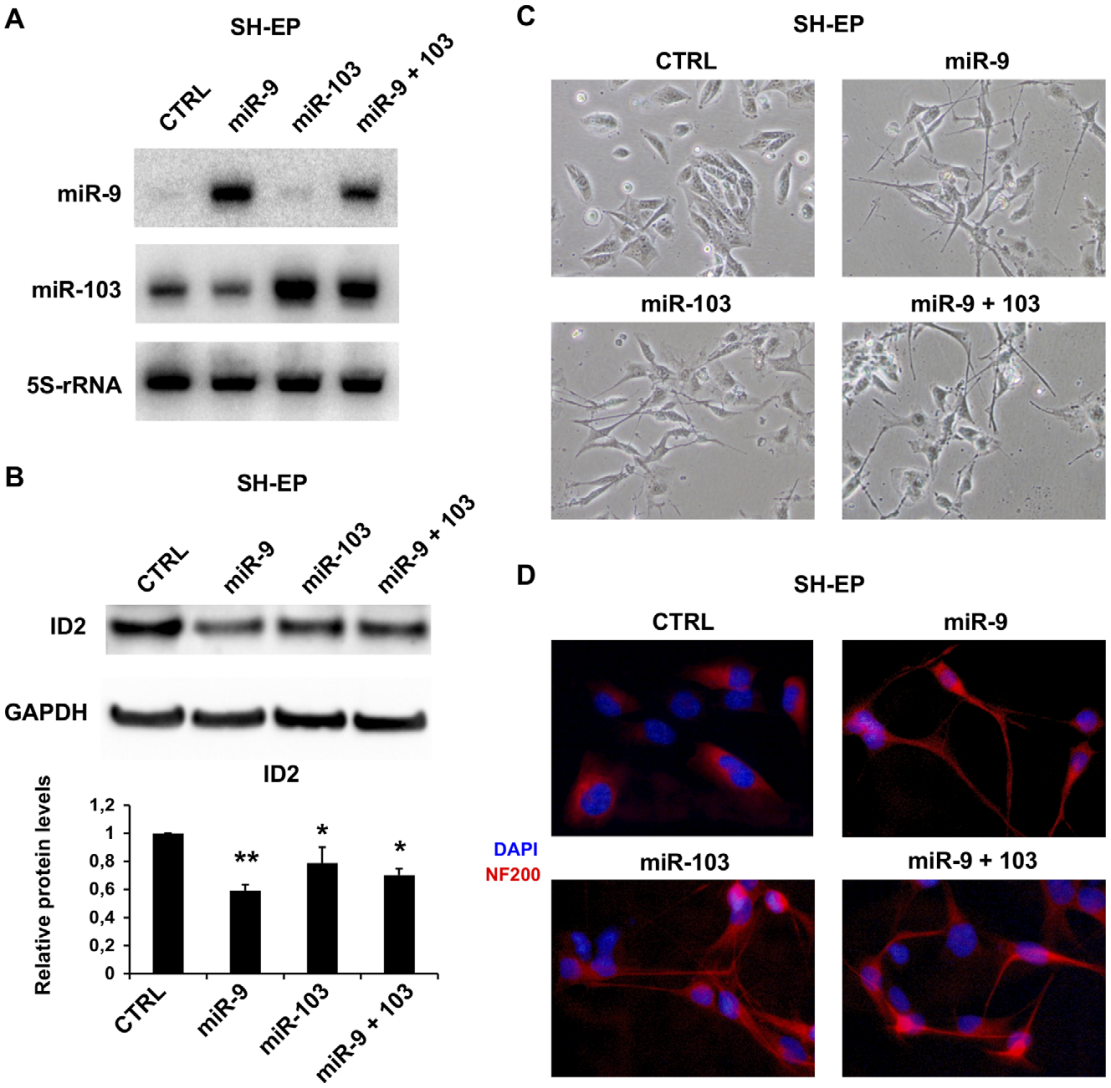
miR-9 and miR-103 trigger neuronal differentiation. (**A**) Northern blotting in SH-EP cells infected with lentiviruses expressing miR-9, miR-103 or empty lentivirus as control. (**B**) Representative immunoblotting of ID2 in SH-EP cells infected with lentiviruses expressing miR-9, miR-103, a combination of the two, or with empty lentivirus as control (CTRL). GAPDH was used as loading control. The histogram displays the relative amounts of ID2 – compared to control cells (mean values ± SD from three independent experiments. *: p-value<0.05; **: p-value<0.01). (**C**) Phase contrast images of SH-EP cells infected with lentiviruses expressing miR-9, miR-103, the two together, or control virus (CTRL). We evaluated the percentage of differentiated cells by counting the number of cells with neurites versus the total number of cells in three microscope fields. Percentages were al follows. CTRL: 0%; miR-9: 84.8±3.2; miR-103: 85.9±2.3; miR-9+103: 80,4±2,7. (**D**) Immunofluorescence staining of the neurofilament heavy polypeptide NF200 (red) in SH-EP cells infected as in panel C. Nuclei were stained with DAPI (4,6-diamidino-2-phenylindole, blue).

The differentiation promoting activity of the 13I domain was mostly evident in SH-EP cells [48], a neuroblastoma cell line similar to neural crest precursors: 13I expression in such cells triggered neuronal commitment and differentiation, with sprouting of multiple, neurite-like processes [22]. We therefore employed this cell line to elucidate the impact of miR-9 and miR-103 on morphological differentiation. Overexpression was achieved by lentiviruses containing miR-9 and miR-103 expression cassettes (Fig. 5A). In accordance with data in SK-N-BE and SH-SY5Y cells – reported in Fig. 3D – both microRNAs, more markedly miR-9, decreased the ID2 level in SH-EP cells (Fig. 5B). miR-9 and miR-103 strongly promoted differentiation, with over 80% of the cells displaying neurite-like processes (Fig. 5C, 5D). Morphological differentiation was accompanied by increased expression and localization of the neurofilament protein 200 (NF200) to neurite-like processes (Fig. 5D). We observed a similar, although weaker, effect after miR-9 and miR-103 ectopic expression in SH-SY5Y cells (data not shown).

**Figure 6:**
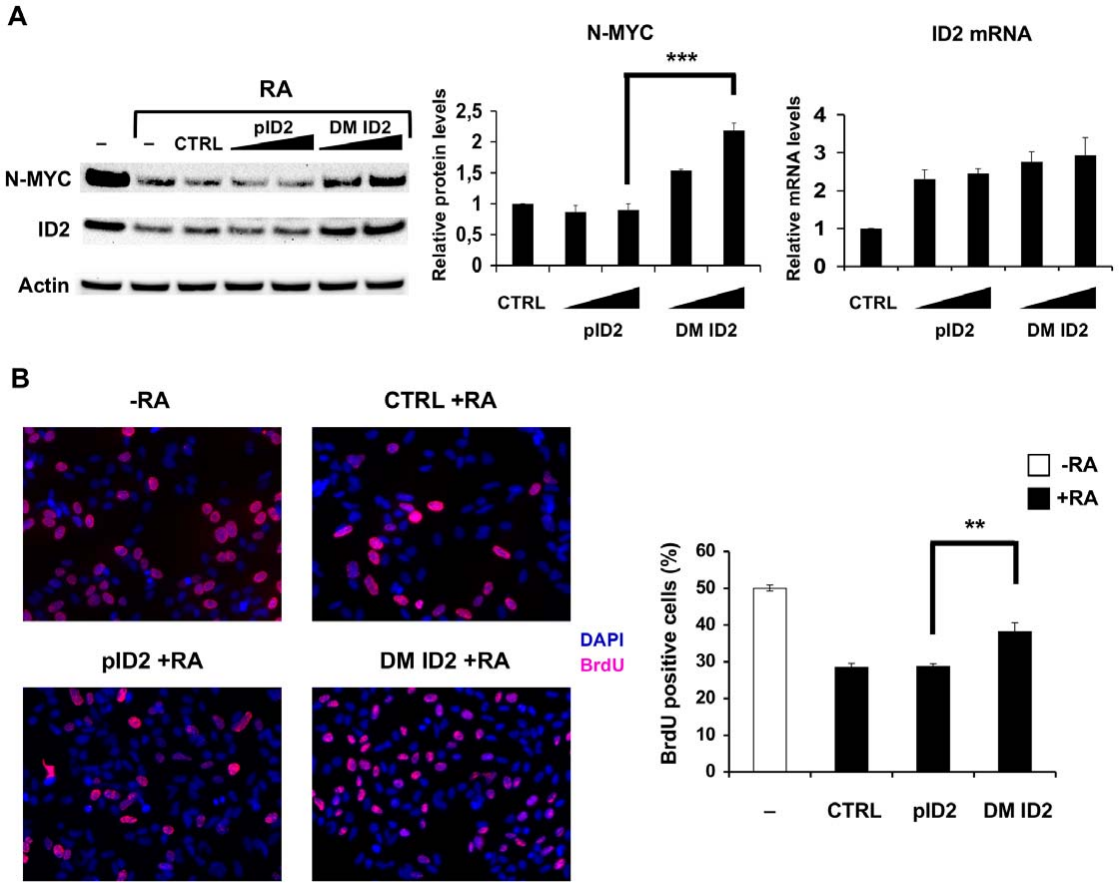
The non-targetable version of ID2 mRNA rescues proliferation rate and N-Myc expression in differentiating SK-N-BE cells. (**A**) Rescue of N-Myc expression. Left panel: representative immunoblotting of N-Myc and ID2 in differentiating SK-N-BE cells (lanes RA) transfected with 1 or 2 µg of vector expressing full-length ID2 cDNA, including 3′UTR (lanes pID2); 1 or 2 µg of the non-targetable version of ID2 cDNA, mutated in both miR-9 and miR-103 recognition sites (lanes DM ID2); the empty vector (CTRL). The symbol – denotes untransfected cells. ß-actin was used as a loading control. Middle panel: histogram displaying the relative N-Myc protein levels – compared to control cells and normalised against actin protein levels (mean values ± SD, from three independent experiments. ***: p-value<0.001). Right panel: the histogram shows the relative ID2 mRNA levels in differentiating SK-N-BE cells transfected with 1 or 2 µg of the above described ID2 constructs – compared to control cells. Values, expressed as means ± SD from three independent experiments, are normalised against *GAPDH* mRNA. (**B**) Proliferation rate rescue. Left panel: immunofluorescence staining of BrdU incorporation (purple) in SK-N-BE cells transfected with 2 µg of wild-type ID2 cDNA (pID2 +RA), 2 µg of the non-targetable version of ID2 cDNA (DM ID2 +RA), or with the empty vector (CTRL +RA). Untreated SK-N-BE cells were also assayed (−RA). Nuclei were stained with DAPI (4,6-diamidino-2-phenylindole, blue). Right panel: quantification of BrdU positive cells. White bar: untreated SK-N-BE cells; black bars: RA-treated SK-N-BE cells, transfected as above. Values, expressed as a percentage of the total cell number, represent means ± SD from three independent experiments. **: p-value<0.01.

These data suggest that post-transcriptional regulation of ID2 by miR-9 and miR-103 has a role in neuroblastoma cell differentiation. To corroborate this idea, we transfected SK-N-BE cells with plasmids expressing ID2 mRNA – wild type or mutated in miR-9 and miR-103 target sites –, treated them with RA and investigated two aspects of their differentiation: the decrease of proliferation rate and the inhibition of N-Myc expression. Both miRNAs are significantly induced upon RA treatment of SK-N-BE cells [29] and restrain their proliferation (Fig. 4A); miR-9 was also shown to inhibit N-Myc expression [29]. If our hypothesis were correct, the non-targetable mutant should rescue proliferation rate and N-Myc expression more efficiently than wild type ID2 mRNA. This is exactly what we observed, as shown in Figure 6. At both concentrations tested, wild type and mutant plasmids produced similar amounts of *ID2* mRNA, further confirming the conclusion that miR-9 and miR-103 target sites do not affect mRNA production (Fig. 6A). On the contrary, only the non-targetable plasmid – unable to bind miR-9 and miR-103 – was able to rescue N-Myc expression (Fig. 6A) and proliferation rate (Fig. 6B) of SK-N-BE cells.

Hence, our findings strongly support the hypothesis that the microRNA-mediated ID2 inhibition has a role in controlling neuroblastoma cell differentiation.

## Discussion

ID2 is a key regulator of neural differentiation, involved in tumorigenesis in the nervous system. Its production is known to be controlled by a variety of signalling pathways affecting ID2 transcription [1], [14], [16], but has not yet been described to be regulated at the post-transcriptional level by miRNAs. We identify miR-9 and miR-103 – upregulated by retinoic acid treatment [29] – as negative regulators of ID2 and differentiation promoting microRNAs in neuroblastoma cells. The possibility that ID2 mRNA was targeted by miR-9 had also been suggested by the bioinformatic analysis of developmentally regulated miRNAs [49]. While several studies have shown that miRNAs bind to coding sequences [31], [50], [51], [52], this is one of the few cases in which the functional role of binding to the coding sequence is demonstrated. Notably, other cases mostly regard transcription factors involved in differentiation control [53]. microRNA targets within the coding sequence appear to cooperate with targets in the 3′UTR for a more significant regulation of protein expression [54]. This prediction is confirmed by our findings that show a clear synergistic effect of miR-9 and miR-103 in SK-N-BE cells (Fig. 3). Moreover, the presence of a binding site for a neural tissue-specific microRNA (miR-9) and a broadly expressed one (miR-103) in the same mRNA would contribute to the tissue specificity of protein expression.

On the basis of our findings, we propose that miR-9 and miR-103 – acting on *ID2* mRNA – directly affect production of the ID2 protein in neuroblastoma cells. As an ID2 decrease favours differentiation, we suggest that the miR-9/miR-103/ID2 module may represent a new element of differentiation control. Concordantly with this possibility, the ID2 decrease in differentiating neuroblastoma cells was accompanied by increased expression of miR-9 and miR-103 and the ectopic expression of the two miRNAs restrained proliferation and promoted neuronal differentiation, reproducing the effects caused by a dominant, ID2 interfering protein [22]. Moreover, an *ID2* mRNA mutated in miR-9 and miR-103 target sites was able to rescue the decrease in proliferation rate and N-Myc expression occurring when neuroblastoma cells are induced to differentiate by retinoic acid. Our hypothesis is coherent with evidence indicating opposite roles of miR-9 and ID2 in neural differentiation control: miR-9 promotes neural fate determination whereas ID2 supports proliferation and self-renewal of neural precursor cells [16], [28], [55]. Notably, miR-9 directly targets a second inhibitor of neural differentiation, REST [35], [56] – which prevents transition from progenitor cells to neurons. This suggests that miR-9 may mediate communication between *ID2* and *REST* mRNAs by acting as a competing endogenous RNA (ceRNA [57]), and allow the coordinate regulation of two key proteins for the switch between proliferation and differentiation in neural cells. We also uncover a novel role for miR-103 in promoting neural differentiation. In accordance with our data suggesting an antiproliferative role for this microRNA, miR-103 was shown to inhibit proliferation of intestinal crypt cells and to be downregulated upon induction of proliferation by IGF-1 [58].

Ultimately, reduction of proliferation and enhancement of differentiation promoted by miR-9 and miR-103 in neuroblastoma cells indicates that these miRNAs may have tumor suppressive properties. This characteristic appears to be context specific since miR-9 and miR-103 were found to be associated to malignancy of breast cancers, by promoting epithelial-to-mesenchymal transition and metastatic potential of breast cancer cells [36], [59]. miR-9 ectopic expression decreased the ID2 level in MCF7 breast cancer cells (not shown), suggesting that the discrepancy, at least in the case of miR-9, is unlikely to result from the inability to target ID2 in breast cancer cells and may be explained by the opposite role of ID2 in the two cell types, at least partly. ID2 is pro-proliferative and pro-metastatic in neuroblastoma cells, whereas it helps in the maintenance of a non-invasive phenotype in breast cancer cells [60]. Moreover, a study reported the hyper-methylation of miR-9 encoding gene promoters in a number of metastatic tumours, indicating an anti-metastatic role of this microRNA [61].

Aside from neuroblastoma, miR-9 is negatively associated to tumorigenic properties in two other neural cancers: medulloblastoma and glioblastoma. miR-9 expression is increased in normal cerebellar tissue versus medulloblastoma specimens, its knock down promotes medulloblastoma cell proliferation, and RA treatment of medulloblastoma cells – similarly to neuroblastoma cells – upregulates miR-9 [62]. miR-9 restrains the tumorigenic potential and is associated to a better prognosis of glioblastoma [63], it suppresses mesenchymal differentiation of glioblastoma cells and inhibits glioblastoma cancer stem-like cell proliferation [64], which is instead promoted by ID2 [16]. Given the established role of ID2 as a neural differentiation inhibitor, an enhanced expression of miR-9 and miR-103 may contribute to promote differentiation of cells from several neural tumours by hindering ID2 production.

Altogether our findings indicate that a miR-9/miR-103/ID2 module may have a differentiation promoting, oncosuppressive function in several neural cancers and may be targeted for restraining their tumorigenic properties.

## Materials and Methods

### Cell culture, expression vectors and transfections

SK-N-BE(2)-C and SH-SY5Y [40] cells were from ATCC (catalogue numbers CRL-2268 and CRL-2266). SH-EP cells [48] were previously described. HEK 293T cells [65] were obtained from A. Levi (IBCN CNR, Roma).

SK-N-BE and SH-EP cells were grown in RPMI-1640 medium (Gibco), SH-SY5Y in DMEM/F12 (1∶1, Gibco), and HEK 293T cells in DMEM – supplemented with 10% foetal bovine serum (EuroClone), L-glutamine and penicillin/streptomycin (Invitrogen) – under standard cell culture conditions. SK-N-BE and SH-SY5Y cells were induced to differentiate with 10 µM all-trans-RA (Sigma-Aldrich).

The plasmid harbouring the human *ID2* coding sequence, but not 3′UTR, under CMV promoter control was previously described [22]. The miR-9 expressing plasmid – containing the human pre-miR-9-3 sequence (from −21 to +93) under control of human snRNA U1 gene expression cassette was as described [29]. To construct the miR-103 expression vector, we amplified by PCR the human pre-miR-103-1 sequence (from −123 to +108, as reported in miRBase) and subcloned it into the snRNA U1 gene expression cassette [29]. Mut ID2 plasmid (harbouring point mutations of the putative miR-9 target site and lacking the 3′UTR) was generated from the *ID2* cDNA expression vector by the QuikChange Site-Directed Mutagenesis kit (Agilent), with the following primers:

5′-CGCATCCCACTATTGTCAGGCTCCACCATCAACGCCCCGGGCAGAACCAGGCG-3′ (forward) and 5′-GCGTAGGGTGATAACAGTCCGAGGTGGTAGTTGCGGGGCCCGTCTTGGTCCGC-3′ (reverse). The introduced mutations are silent, except for the substitution of serine with arginine at position 85. The luciferase reporter plasmid of *ID2* 3′ UTR was obtained by PCR-amplification and cloning of the human *ID2* 3′ UTR into pRL-TK (Promega). The reporter plasmid carrying a deletion of the putative miR-103 binding site (ΔmiR-103) was generated from the *ID2* 3′ UTR reporter by inverse PCR with the following oligonucleotides: 5′-GAGTTTTCTTGTATAGTGGC-3′ (forward) and 5′-GATCCTTCTGGTATTCACGC-3′ (reverse). The ID2 expressing plasmid harbouring full length cDNA (pID2) – including the 3′UTR – was generated by ligation of ID2 coding sequence to wild-type 3′UTR and cloning into pCDNA™ 3.1(+) (Invitrogen). The double mutant ID2 expressing plasmid (DM ID2) – harbouring full length cDNA mutated in miR-9 and miR-103 target sequences – was generated by ligation of Mut ID2 coding sequence (mutated in the miR-9 binding site) to the 3′UTR sequence carrying the miR-103 binding site deletion (ΔmiR-103), and cloned into pCDNA™ 3.1(+). All constructs were verified by sequencing.

Expression plasmids were transfected into SK-N-BE and SH-SY5Y by Lipofectamine Plus Reagent (Invitrogen), and into 293T and MCF7 cells by Lipofectamine 2000 (Invitrogen), in OPTI-MEM I medium (Gibco). A plasmid producing a 21 nucleotide long RNA, bearing no homology to any known miRNA or mRNA sequence in human [29], was used as control.

miR-9 and miR-103 expressing lentiviral vectors were obtained by subcloning their expression cassettes into pRRLcPPT.hPGK.EGFP.WPRE [66]. Infective particles were produced and neuroblastoma cells were infected by standard methods. An empty lentivirus was used as control. The efficiency of lentiviral transduction was evaluated by the presence of EGFP by fluorescence microscopy (data not shown).

### Luciferase reporter assay

Cells were cotransfected with control or microRNA over expressing plasmids, wild-type or mutated *ID2* 3′UTR Renilla luciferase reporter plasmids, and the Firefly luciferase reporter plasmid pGL3 (Promega). Cells were harvested 48 h after transfection. Firefly and Renilla luciferase activities were measured by the Dual-Luciferase Assay (Promega). All assays were performed in triplicate in three independent experiments.

### Immunoblotting and immunohistochemistry

Whole-cell protein extracts were prepared from cells lysed in RIPA buffer. Samples were separated through SDS-PAGE gels, transferred to Hybond ECL membranes (GE Healthcare), and treated with appropriate antibodies. Staining was performed by SuperSignal Chemiluminescent Substrate (Pierce). ImageJ software (rsbweb.nih.gov/ij/) was used for densitometric analysis; western blot quantification was normalized against loading controls. Immunohistochemical detection was performed as described [22].

ID2, N-Myc and GAPDH antibodies were from Santa Cruz Biotechnology (sc-489, sc-56729 and sc-32233, respectively), Actin and Neurofilament 200 (NF200) antibodies were from Sigma (A2066 and N0142), and Vgf antibody was as described [45], [47]. Horseradish peroxidase secondary antibodies were from Chemicon and Protein A peroxidase from Sigma.

### Cell Proliferation Assay

Cells were transfected with expression plasmids or control plasmid, treated or not with RA; cell proliferation rate was analysed by the BrdU assay as described [29].

### Northern blot analysis

The northern blot analysis was carried out as described with minor modifications. Total RNA was extracted with TRIzol Reagent (Life Technologies), fractionated on 10% poly-acrylamide gel in 1× TBE, 7 M Urea and transferred onto Amersham Hybond-NX nylon membrane (GE Healthcare). DNA oligonucleotides complementary to the sequence of mature miR-9, miR-103 and to 5S-rRNA (5′-AGACGAGATCGGGCGCGTTCA-3′) were ^32^P-labelled and used as probes.

### Analysis of ID2 mRNA expression by quantitative RT-PCR (qRT-PCR)

1 µg of DNA-free RNA was reverse-transcribed using SuperScript ® III First-Strand Synthesis SuperMix (Invitrogen). In order to quantify the expression of ID2 mRNA, 20 ng of each generated cDNA were amplified in triplicate in the presence of QuantiTect SYBR Green PCR Master Mix (Qiagen) and 0,5 µM ID2 mRNA specific primers: 5′-CAGAACAAGAAGGTGAGCAAGATG-3′ (forward); 5′-CACAGTGCTTTGCTGTCATTT-3′ (reverse). Thermal cycle conditions were the following: 15 min of initial setup at 95°C, followed by 40 cycles at 94°C for 15 s, 55°C for 30 s and 70°C for 30 s. Data were calculated with 7500 Software v2.0.5 (Applied Biosystems) by the ΔΔCT method and expressed as relative quantities after GAPDH normalization.

### Statistical analysis

Data are presented as means ± standard deviation, and unpaired, two-tail, Student's *t*-test was used to compare groups for independent samples. A p-value<0.05 was considered significant.

## Supporting Information

Figure S1 **The** ***ID2*** **mRNA levels were not altered by miR-9 and miR-103 overexpression.** ID2 mRNA levels were evaluated by qRT-PCR upon miRNA ectopic expression in SH-SY5Y cells. Values are relative to control cells transfected with an unrelated 21 nucleotide long RNA (CTRL). RNA from cells transfected with a muscle-specific miRNA was also analysed (column miR-206).
(PDF)

Figure S2 **ID2 ectopic expression in RA-treated SK-N-BE cells inhibits the expression of the differentiation marker VGF.** Immunoblotting of VGF and ID2 in SK-N-BE cells ectopically expressing ID2 or the empty vector (CTRL), either untreated (lane – RA) or treated for three days with retinoic acid (lanes +RA). GAPDH was used as a loading control.
(PDF)
