# Supplementary figures and images for: A New Module in Neural Differentiation Control: Two MicroRNAs Upregulated by Retinoic Acid, miR-9 and -103, Target the Differentiation Inhibitor ID2
Source: PLoS One. 2012 Jul 25;7(7):e40269. doi: 10.1371/journal.pone.0040269 (PMC3405103; doi:10.1371/journal.pone.0040269)

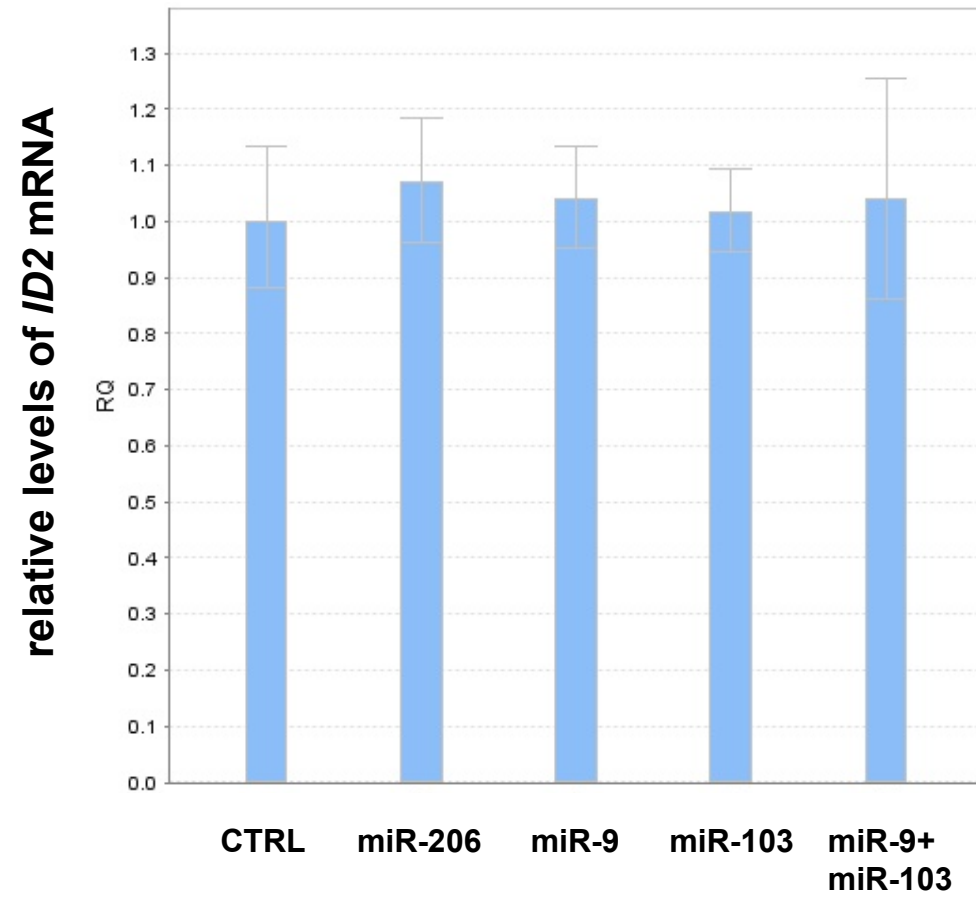

Supplement: Figure S1 — The ID2 mRNA levels were not altered by miR-9 and miR-103 overexpression. ID2 mRNA levels were evaluated by qRT-PCR upon miRNA ectopic expression in SH-SY5Y cells. Values are relative to control cells transfected with an unrelated 21 nucleotide long RNA (CTRL). RNA from cells transfected with a muscle-specific miRNA was also analysed (column miR-206). (PDF) [file pone.0040269.s001.pdf]

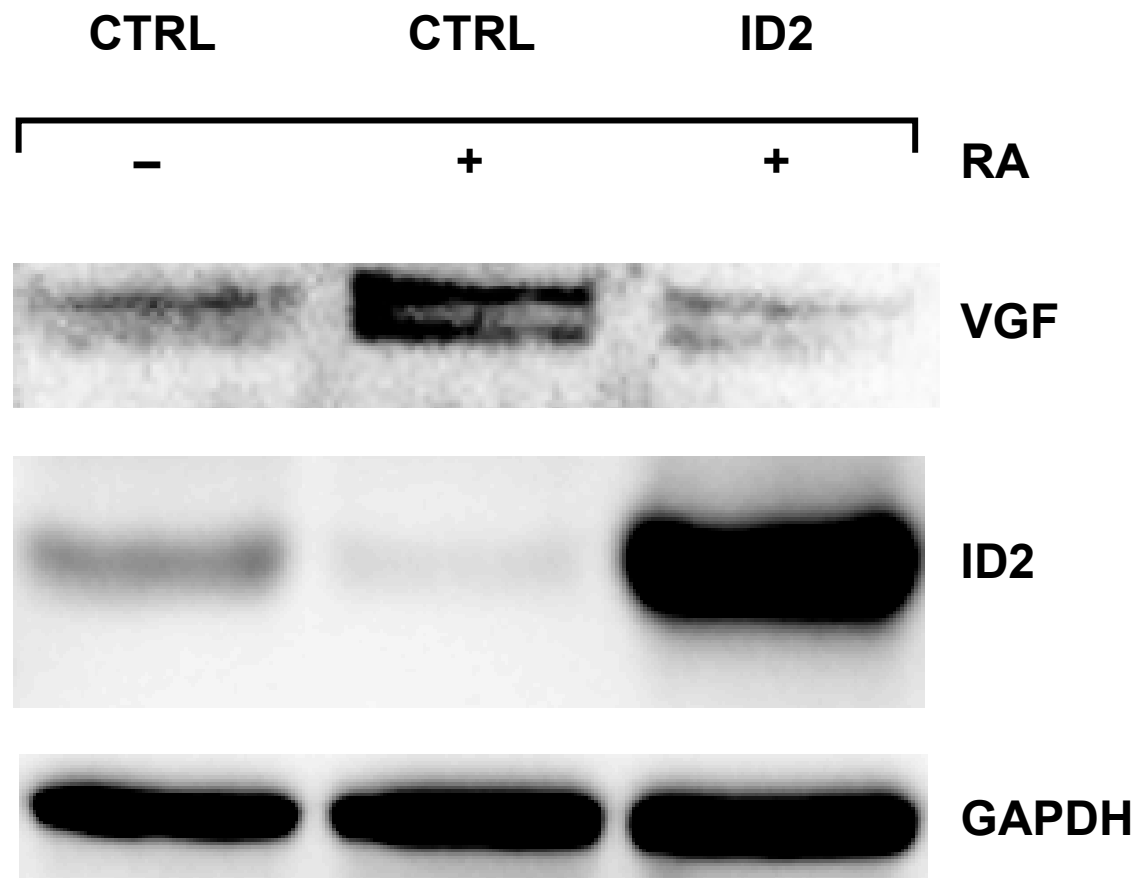

Supplement: Figure S2 — ID2 ectopic expression in RA-treated SK-N-BE cells inhibits the expression of the differentiation marker VGF. Immunoblotting of VGF and ID2 in SK-N-BE cells ectopically expressing ID2 or the empty vector (CTRL), either untreated (lane – RA) or treated for three days with retinoic acid (lanes +RA). GAPDH was used as a loading control. (PDF) [file pone.0040269.s002.pdf]
